# A Clinical Treatise on the Diseases of the Heart, Preceded by Original Researches on the Anatomy and Physiology of That Organ

**Published:** 1836-04

**Authors:** 


### Art. IV.

*Traité Clinique des Maladies du Cœur, precédé de Recherches nouvelles sur l'Anatomie et la Physiologie de cet Organe.* Par J. Bouillaud, Professeur de Clinique Médicale à la Faculté de Médecine de Paris.—*Paris*, 1835. Deux tom. 8vo. pp. 534, 632.

*A Clinical Treatise on the Diseases of the Heart, preceded by original Researches on the Anatomy and Physiology of that Organ.* By J. Bouillaud, Clinical Professor to the Faculty of Medicine at Paris.

Notwithstanding the zeal and assiduity with which the physiology and diseases of the heart have of late years been studied, and the number of individuals of high and acknowledged talent engaged in such investigations, the subject is yet far from being exhausted; nor is our knowledge of it perhaps even yet brought up to the level of that possessed concerning many less important organs. Several points of great doubt and difficulty still agitate the medical world in regard to it; and, under these circumstances, it was with much satisfaction that we observed the announcement of the present work of M. Bouillaud, an author already so favorably known by his labours as editor and joint-author of M. Bertin's Treatise on the Diseases of the Heart, which appeared about eleven years since. The present is by no means to be looked upon as a republication of the former work: it may be said, however, to contain all that was really valuable in it, with an immense accession of additional facts; the practical part bearing the stamp of increased experience, and the theoretical portion, of matured judgment and more correct generalization. Some of the hypotheses which, in the former work, were put forward in too absolute and unqualified a manner, have here been to a certain degree modified, and the range of their application somewhat limited.

The foundation of our present knowledge of the diseases of the heart and great vessels appears to have been laid by Lancisi, Valsalva, and Albertini. Morgagni followed up the subject with great success; and about the same period Senac brought out his valuable Treatise on the Structure and Diseases of the Heart. When Lancisi pointed out the fact, that oppressions of the chest, and asthmatic affections, instead of being idiopathic, as formerly supposed, originated often in disease of the heart, he did a signal service to pathology. This idea was not lost on Morgagni, who gave it additional development and illustration, though its full importance was scarcely appreciated till the time of Corvisart. The last-named writer's Essay on the Diseases of the Heart forms an era in respect to this class of affections: his new division of aneurism of this organ, though not sufficiently comprehensive, nor altogether correct, had the merit of concentrating attention on the subject, and paving the way for the subsequent more accurate investigations of Bertin and others. His account of pericarditis, of vegetations and indurations of the valves, and the consequent contraction of the cardiac orifices, together with the revival of Avenbrugger's invaluable method of percussion, fully establish his claims to a distinguished place amongst the successful investigators of this class of diseases. He was speedily followed by Burns in this country, Testa in Italy, and Kreysig in Germany. Bertin, in a succession of memoirs presented to the Academy of Sciences, in 1811, and subsequently, brought forward some very original views as to the true nature and mode of formation of aneurisms of the heart, as they used to be called; and, not very long after, Laennec threw a brilliant light on the diagnosis of diseases of the heart, as well as of the other organs contained within the cavity of the thorax, by his happy discovery of auscultation. In 1824 appeared M. Bertin's Treatise on the Diseases of the Heart, mentioned above. Dr. Hope's work on the Diseases of the Heart and Great Vessels, with its ingenious investigations as to the motions and sounds of the former, appeared in 1832, and served to give a fresh impulse to this branch of medical science, which has possessed during the last few years a greater number of indefatigable and able cultivators than almost any other department of medicine. The names of Andral, Louis, Turner, Magendie, Williams, Corrigan, Pigéaux, Carlisle, Stokes, and many others, will occur to our readers, and justify the above assertion.

M. Bouillaud founds his claims to being considered a useful labourer in this field, on the following grounds: first, that he has been instrumental in giving additional development to many observations made by preceding enquirers in relation to this class of disorders, and thrown the light of analysis on many of the complex affections heretofore vaguely designated under the general terms of aneurism or organic lesion of the heart. It is thus, for example, that he claims to have demonstrated that the peculiar collection of symptoms, referred by Corvisart and his followers to hypertrophy or to aneurism of the heart, is in most cases merely an effect of certain lesions of the valves of this organ; lesions, of which the aneurism and hypertrophy should themselves also be viewed only as consequences.

Secondly, he conceives he has thrown a new light on acute inflammation of the pericardium, and that henceforward pericarditis, in relation to its causes, symptoms, progress, and termination, may be considered fully as well understood by us as pleurisy itself; a conclusion in which, we apprehend, many of our readers are not yet prepared to acquiesce; and which, indeed, the cases in the subsequent part of the work, (and we allude especially to those where an attempt has been made to contrast, and mutually to distinguish, inflammations of the internal and of the external lining of the heart,) scarcely bear out. The failure is most conspicuous in those instances of disease where the effusion is extremely limited in quantity. As to the etiology of pericarditis, he believes that, in a vast proportion of instances, the disease is connected with rheumatism; a source which, in a more limited sense, has long been recognized by practitioners here, though probably few will agree with our author in viewing it as one of the ordinary concomitants of acute articular rheumatism, or admit that it exists in at least one half of all such cases. Indeed, even in France, M. Bouillaud is considered to carry the matter rather too far in this respect; and Fouquier, Marjolin, and others of great experience, protest against such an extreme degree of latitude being given to a cause of which, in a more confined circle, they fully acknowledge the influence. In the treatment of this disease he thinks he has introduced a valuable improvement, in his mode of very copious and frequently repeated bleedings, in consequence of which recovery has been effected in a very great proportion of his cases; and death, which in Corvisart's time was the rule, has now, as he expresses it, become the exception.

Thirdly, he conceives that he has conferred on medical science an important benefit by his investigation of the inflammation of the sero-fibrous tissue lining the internal surface of the heart's cavities; a lesion which, prior to his enquiries, had excited so little notice as not even to have been thought worthy of name. On this disease, on account of its situation, he has bestowed the appellation of *Endocarditis;* and to his description of it he points with confidence as being at once the most original and valuable portion of his book; conceiving a knowledge of it to be of first-rate importance, as well on account of the frequency of its occurrence, which is much greater, he thinks, than most practitioners are aware of, as on account of the serious organic lesions of the cardiac orifices and their valves, as well as of the subjacent muscular structure, to which, if suffered to pass into its chronic stage, it tends to give rise. Its chief cause, like that of inflammation of the external covering of the heart, with which it so often coexists, is, he is satisfied, of a rheumatic nature; for it is a very frequent concomitant of acute rheumatism of the joints, and exposure of the body to cold when in an over-heated state is one of the most frequent conditions under which it arises. The obscure cases of arthritic asthma, as it was called, to be met with in the older writers, were, he believes, neither more nor less than instances of this affection in its advanced and chronic form.

The practical part of the work is prefaced by a detailed investigation of the anatomy and physiology of the heart, with a full exposition of its sounds, normal as well as abnormal, as also of those of the arteries, and a long discussion of the various theories of their production hitherto advanced. The pathological portion of the book is divided into two parts, the first of which consists of general considerations as to the anatomical characters and the precise seat of the various diseases of the heart; their physiological characters and their diagnostics; their causes; their nature and classification; their progress, duration, and probable termination; their treatment; and finally their complications, as well with each other as with diseases of other organs.

In the second part each affection of the heart is considered individually, and in the order of its place in the classification which M. Bouillaud has adopted; a classification which, though it appears to him better suited than any hitherto proposed to the actual state of our knowledge, he is far from considering as perfect. His first class comprehends such diseases as consist essentially in the lesion of the molecular actions of the heart ("actes intimes et moléculaires"), such as secretion, nutrition, &c. and consequently comprises acute and chronic inflammation *(Pericarditis, Endocarditis, Carditis;)* increase and diminution of secretion *(active Hydropericardium;)* increase and diminution of absorption, *(passive Hydropericardium, Hydro-pneumocardium;)* increase and diminution of the nutrition of the heart, *(Hypertrophy, Atrophy.)*

This second class consists of the neuroses of this organ, which he subdivides into those characterized by increase of action, hyperdynamia, (palpitations, spasm,) diminished action, adynamia, (swooning, syncope, &c.) irregularity of action, ataxo-dynamia, (inequality, irregularity, or intermittence of the pulsations.)

His third class comprises physical and mechanical lesions of the heart, (wounds, ruptures, dilatation, or contraction of its cavities or orifices, displacements,) &c. The fourth or last class consists of congenital malformations; whilst in an appendix there is an account of the coagulation of the blood within the cavities of the heart; or, in other words, of polypous concretions formed during life.

For the minute details of the cases, of which about two hundred are given, a great proportion of which are his own, he thinks no apology necessary; their increased length and fulness, as compared with those of most former writers, being the natural consequence of the extended state of our knowledge of these diseases. In regard to method, accuracy, sufficiency, or relevancy of detail, the cases in many late French works cannot be read and compared with the meager and hasty sketches of some of our own writers without exciting rather humiliating reflections, and affording ample conviction of the fact that the art of case-taking is as yet far from having been brought to the same general degree of perfection here; an inferiority which may in part be attributed to the little responsibility till lately imposed on the great majority of our clinical students, as well as to the somewhat discreditable fact that cases are too often taken with no other view than to swell the pages of a book got up under the influence of a desire for temporary notoriety and worldly advancement, rather than under the genuine inspiration of a love of science. To this, however, we need scarcely add that there are very many honorable exceptions.

In the present article, we shall only be able to review the first portion of M. Bouillaud's work; and we shall commence by noticing some of the more important points alluded to by our author in relation to the anatomy and physiology of the heart. After deprecating the unqualified assertion of Corvisart and Laennec as to the utter impracticability of accurately describing the heart, or of ascertaining the relative proportions of its parts with any thing approaching to mathematical precision; as well as that of Cruveilhier, as to the impossibility of determining the precise limit which separates the normal from the abnormal state; he endeavours to shew that we may, by numerous observations and carefully repeated comparisons, attain to a very adequate notion of the mean volume and mean weight of the organ. He reminds us that here, as in all other departments of medicine, the *approximative* calculus is the only one with which we have to do. He has accordingly gone through a very laborious series of measurements of the size of the several cavities of the heart in a great variety of subjects, differing from each other as to age, sex, &c.; as well as of the absolute and relative dimensions of the orifices of communication, their valves, &c.; and to the results at which he has arrived we shall presently recur.

A knowledge of the anatomical relation of the anterior inferior portion of the lungs to the heart is very important in regard to diagnosis, for it points out to us the natural limits of the dull sound on percussion of the cardiac region in the healthy state. The extent of this dulness corresponds to that portion of the pericardium and heart which remains uncovered by the spongy and resonant tissue of the lung: any morbid enlargement of the one, or effusion into the other, increases the exposed surface to an extent which is accurately indicated by the increased dulness. Our author, however, seems to have overlooked two possible sources of error. The augmented dulness on percussion, though a very valuable sign, is yet not an infallible proof of the existence of the lesions above alluded to; for the interposition of a portion of hepatized lung, or of any tumour between the pericardium in the front of the chest, or a partial pleuritic effusion confined by false membranes, and local adhesions, would give rise to the same physical phenomenon; whilst an emphysematous state of the lung in the same situation might cause an error of an opposite kind, as it would mask in some degree the existence of an enlargement of the heart or the presence of a fluid distending the pericardium. "The anterior inferior edge of the right lung in the natural state projects a little over the right side of the pericardium and corresponding portion of the heart; whilst the left lung, advancing similarly over the left part of the pericardium, covers a considerable extent of the left cavities. The portion of the pericardium which is ordinarily left uncovered is that corresponding to the two-thirds of the anterior surface of the right ventricle: it presents the figure of a lozenge, and is from an inch and a half to two inches square." In some very rare cases, however, almost the whole anterior surface of the heart is covered by the lung.

The tortuous disposition of the muscular fibres of the heart, which baffled Steno and many subsequent anatomists, has been more successfully investigated in later days by Wolff, Duncan, and Gerdy, who have shewn that the parietes of the ventricle consist of several layers differing in number for each ventricle. Of these layers there are, according to M. Gerdy, six in the left ventricle, and only three in the right; and hence the comparative thinness of the walls of the latter. The fibres of the external layers run obliquely from above downwards, from before backwards, and from right to left; the middle layers take in all respects the opposite directions; and the deep-seated ones, which by their union form the fleshy columns projecting into the interior of the cavities, are for the most part longitudinal. The most superficial layers, passing along the apex, occupy the entire circumference of the ventricles, whilst the others diminish in length and breadth in proportion as they follow a deeper course; and hence it is that the ventricles are so much thicker at the base than at the point of the heart. All the fibres, whatever may be their disposition in other respects, turn upon themselves in such a manner at the middle point as to form a species of loop, the convexity of which looks towards the apex of the organ: the more superficial the fibres are at the one extremity, the deeper seated do they become at the other: thus the most external fibres, for example, become before their termination the most internal, in consequence at once of their having been reflected in the manner just described, and also of having traversed the thickness of the ventricle. The extremities of these loops are invariably inserted at the base of the heart around the circumference of the auricular and arterial orifices of the ventricles, either immediately or, in a smaller number of instances, by the tendons attached to the auriculo-ventricular valves, (chordæ tendineæ). The auricles, which are likewise of a very complicated structure, are, according to the same authority, composed of two muscular layers, the one external, the other internal. In the right auricle the muscular tissue, being less abundant than in the left, leaves occasional intervals between its fibres where the internal and external membranes of the heart are in almost immediate contact; and this proximity, which is best seen in the hearts of individuals in whom the right auricle has been considerably dilated with thickening of the muscular fibres, is turned to account by M. Bouillaud in a subsequent part of his work, as helping to explain the frequent coexistence of inflammation of the internal lining of the heart with that of the pericardium. The conclusions which have been arrived at by Mr. Carlisle as to the structure of the heart approach very nearly to those of M. Gerdy, whilst M. Filhos, on the other hand, still calls in question the continuity of the external with the deeper seated fibres; it appearing to him that, after turning from right to left and from above downwards in a spiral direction near the apex of the ventricle, they terminate in a well-marked raphé, from which the internal fibres likewise take their origin. The direction of the fibres of the right ventricle, moreover, he conceives to differ very materially from those of the left, forming semi-ellipses, of which the extremities are directed upwards and the concavity downwards. It appears to us, however, from the examination of the heart of a large animal, the fibres of which had been rendered more easy to disentangle by long continued boiling, that the opinions of the former of these observers are more correct than those of M. Filhos; and such seems likewise to be the conviction of M. Bouillaud.

The columnæ carneæ are much more numerous, but smaller, in the right ventricle than in the left, though the contrary has been inadvertently stated by Laennec. The muscular pillars into which the chordæ tendineæ of the valves are inserted are, according to our author, specially destined to raise the valves from the flattened position against the sides of the ventricles, and thus to effect the closure of the auriculo-ventricular orifices; but he is here in contradiction with Bichat and other celebrated anatomists; and it seems to us that, in his attempts to make out this point, he is not very clear or satisfactory.

The chordæ tendineæ are not the only examples of fibrous or albuginous tissue within the heart; for it exists also in the whitish zones or rings at the base of the valves, forming the contour of the orifices; also within the duplicature of the valves themselves; and it is this structure especially which is so frequently the seat of cartilaginous and osseous transformations. The existence of such a tissue here, taken conjointly with the sero-fibrous nature of the envelope of the heart, enables us the more readily to understand why rheumatism should so frequently make its attack on this organ. The internal lining of the heart, or endocardium, as M. Bouillaud names it, and which is to the inner surface of the organ what the serous layer of the pericardium is to the outer, has been too slightly attended to by anatomists and pathologists; though, according to him, its diseases are perhaps even more frequent than those of the pericardium itself. These have been hitherto almost entirely overlooked or misunderstood in the acute stage, and thus permitted to pass into the chronic; and he conceives that he is guilty of no exaggeration in asserting that the affections of the endocardium are by far the most frequent starting-point of those organic lesions, as well of the valves as of the cavities of the heart, formerly, and even still at the present day, confounded together under the vague term of aneurism. The endocardium, in its natural state, is whitish, semitransparent, and pellucid, like the serous membranes; and, like them too, it readily receives by imbibition the reddish staining so often observable under certain conditions in the interior surface of the heart. Its thickness does not exceed that of the most attenuated serous membranes, the arachnoid, for example; and it is somewhat more delicate in the right cavities than in the left; whilst, at the arterial and auriculo-ventricular orifices, it is obviously somewhat thicker than elsewhere: it is in these portions especially that the effects of chronic inflammation take place; and amongst these are to be reckoned the thickening, not merely of this membrane itself, but also of the subjacent cellular and fibrous tissues. Under this influence it sometimes acquires the consistence of a fibrous membrane; and we may then often discover several successive layers placed one over the other, as is so frequently the case after inflammation of the serous membranes, to which this bears such an analogy; whilst, at the same time, it is obvious, on careful inspection, that only one of these, namely, the deepest-seated, really constitutes the membrane in question, the other layers being merely organized pseudo-membranous matter. These false membranes, in their organized state, for the most part present themselves only in a partial or disseminated form, constituting the whitish patches analogous to those so often observed on the pericardium. In some cases, again, the apparent thickening of the endocardium depends on hypertrophy of the subjacent cellular tissue. In the healthy state, this membrane adheres so firmly to the cellular tissue that it can be detached only in very small shreds; whilst, in certain morbid conditions, on the contrary, we can easily raise it in considerable patches. On the valves, its adhesion is particularly firm, and it is scarcely possible to separate their two layers from one another, or from the fibrous tissue contained within them, save at their base, where they separate to receive the tendinous ring which borders the orifices. At this point, too, as well as in that already indicated, the external and the internal membrane of the heart are almost in contact with one another, and this forms an additional explanation of the frequent coincidence of external and internal inflammation of the heart. In its normal state, the endocardium is perfectly smooth and polished, but, by certain morbid affections, may be rendered rough and uneven; and, when in this condition, the friction of the column of blood over it will necessarily be increased.

To M. Bouillaud's original researches as to the weight and volume of the heart in general, the absolute relative dimensions of its walls, cavities, orifices, and valves, we have already alluded. He conceives that Laennec's assertion as to the equality of the several cavities of the heart in the natural state is not strictly correct: for, in most subjects, he has found that the cavities of the right side exceed in capacity those of the left; as also that the auricles usually surpass the ventricles; and, with regard to the ventricles themselves, it has appeared to him that the difference is greater than could fairly be attributed to the distention of that of the right side, by the accumulation of blood in it during the last struggle of nature; and Legallois has observed a similar difference in the lower animals, even when killed by hemorrhage, though it is somewhat less marked than in those which have been put to death by asphyxia, or any other means that, like it, by embarrassing the circulation, favour such accumulation.

With regard to M. Cruveilhier's assertion, that the existence of hypertrophy of the left ventricle should be admitted whenever its walls have attained to the thickness of seven or eight lines, M. Bouillaud very properly points out a deduction which is to be made in regard to these cases, when, a state of general anæmia or marasmus having existed, the heart, though really atrophic, has, in order to accommodate itself to the diminished quantity of blood passing through it, become contracted on itself, and thus given to its parietes an apparent thickness of eight or even of ten lines; and, when the author just alluded to makes five lines the lowest limit of hypertrophy of the right ventricle, (though, leaving this peculiar case out of view, *three* lines would, according to M. Bouillaud, have been nearer the mark,) a similar reservation is applicable. The mean weight of the healthy heart in the adult is stated by Cruveilhier at six or seven ounces, which approaches pretty closely to that assigned it by our author.

M. Lobstein, of Strasburgh, attributes the following mean weight and dimensions to the healthy adult heart, which in some particulars differ considerably from those given above, as well as from those attained to by M. Bouillaud, afterwards to be mentioned.

| | |
|---|---|
| Weight of heart . . . . . . . . . . . | 9 to 10 ounces. |
| Length from base to apex . . . . . . . | 5 inches 6 lines. |
| Breadth at the base . . . . . . . . . . | 3 inches. |
| Thickness of walls of left ventricle . . . | 7 lines. |
| Ditto at a finger's breadth above the apex . | 4 lines. |
| Thickness of walls of right ventricle . . . | $2\frac{1}{4}$ lines. |
| Ditto at apex . . . . . . . . . . . . | $\frac{1}{2}$ line. |
| Thickness of right auricle . . . . . . . | 1 line. |
| Ditto of left auricle . . . . . . . . . | $\frac{1}{2}$ line. |

M. Bouillaud is of opinion that the hearts from which some of these deductions were made could not have been in a natural condition; for the thickness of the right auricle, in place of being double that of the left, as in the above table, is really generally inferior to it; and a heart weighing ten ounces, if belonging to an individual of an ordinary size, must have been hypertrophied. M. Bouillaud weighed the hearts of thirteen subjects, in which, from the general habit, the previous state of health, and the mode of death, there was every reason to think they were in tbe natural state. The mean of all these weights was eight ounces three drachms; the maximum about eleven ounces, but that was in an individual of colossal size and very strong constitution; the minimum was but six ounces two drachms, but it was the heart of a boy of only sixteen years of age, whose body had not yet attained to its full degree of development. From these data he is led to fix the mean weight of the heart in the adult, from the twenty-fifth to the sixtieth year, at from eight to nine ounces. The weight of the heart in women is generally less than that in men; he has not, however, yet ascertained in what exact proportion. In his investigation, it is to the *absolute* weight of the heart he has alone had regard; but he alludes briefly to the experiments of M. Jules Pelletan, as to the difference of its *specific* gravity in different individuals, proving that the weight and the bulk are by no means always in a direct ratio.

With regard to the dimensions of the heart, out of seven cases examined by M. Bouillaud, the mean circumference, measured round the base of the ventricles, was eight inches nine lines; the mean length of the heart in nine cases, measured by a line joining the root of the aorta to the apex, three inches seven lines and a third; the mean breadth at base, measured in eight subjects, was three inches seven lines and a half; the mean thickness of the walls of the left ventricle at the base, six lines and a half; maximum, eight lines, minimum, five lines; the mean thickness of the right ventricle at the base, two lines and three fifths; maximum, three lines and a half, minimum, one and a half. Thus, in adults, the mean thickness of the left ventricle may be stated, in round terms, at seven lines, and that of the right at two lines and a half; or in the proportion of nearly five to two. In infancy no such great disproportion exists; and in the fœtus the walls of the two ventricles are, as is well known, nearly equal in thickness. The mean thickness of the left auricle, measured in four subjects, was found to be a line and a half; the maximum two lines, and minimum three quarters of a line: whilst the mean of the right auricle was only one line; the maximum one and a half, and the minimum half a line; the parietes of the left thus exceeding that of the right by about one-third.

The capacity of the ventricles was not very accurately ascertained, but it may be roughly stated that, in their natural condition, they are of a size capable of holding a hen's egg; the left, however, being obviously a little inferior to the right, as above stated. The right auricle also appears generally slightly to surpass the left in magnitude.

The mean circumference of the left auriculo-ventricular orifice, measured in three subjects, was three inches six lines and a third; that of the right, three inches ten lines. The mean circumference of the ventriculo-aortic orifice, as measured in four subjects, was two inches five lines and a half. The mean circumference of the ventriculo-pulmonary orifice, in the same individuals, was two inches seven lines and three quarters. Thus, it appears that the auriculo-ventricular orifices usually surpass the openings of the aorta and pulmonary artery; and that, of the two latter, the circumference of the second generally excels the first in a slight degree.

M. Bouillaud next proceeds to detail his measurements of the length and thickness of the several valves of the heart, which we have not room to notice at length here. He also gives a long list of the weights and measures of hypertrophied and atrophied hearts, for which we must refer the reader to the work itself. The weight of the largest hypertrophied heart he has met with was more than thrice the mean normal weight, and five times that of those in a state of extreme atrophy. The largest heart mentioned by M. Bouillaud, and which he compares to that of a calf, though taken from a woman of an extremely slight make, was found to weigh 688 grammes, or about a pound and a quarter. M. Lobstein speaks of one which weighed about two pounds, that is, 312 grammes more than the above; but it is possible that it was weighed previously to removing the clots of blood within it, which occasionally amount to about one-third of the gross weight. Of six hypertrophied hearts, the greatest circumference at the base was twelve inches; the greatest length, five inches three lines; the greatest thickness of the left ventricle, one inch one line; that of the right, four lines and a half. Of three cases of hypertrophy of the left auricle, the maximum thickness was only two and a half lines, or about a line more than in the normal condition; whilst in the right auricle, in the same morbid state, a somewhat greater difference was occasionally found. Of concentric hypertrophy, one of the most remarkable examples given was one in which the heart weighed about twelve ounces, and yet the right ventricle would scarcely have held a pigeon's egg, or the left have admitted the fore-finger into its cavity; whilst in another case of hypertrophy, which was of the excentric or true aneurismal kind, the heart weighed about fifteen ounces, the left ventricle was capable of holding an ostrich's egg, and the right was very nearly equally capacious.

Of atrophy of this organ, one of the most striking instances met with was in a woman who had died in a state of extreme emaciation from scirrhus pylori: the heart, which appeared much shriveled, weighing only about four ounces (135 grammes); the left ventricle would hardly admit the little finger, and the right was very little larger.

As the heart of the human subject, when in an extreme state of hypertrophy, has sometimes been compared to that of an ox, the weight and some of the dimensions of the latter have, as a matter of curiosity, been inserted by M. Bouillaud, in a note. After it was separated from the great vessels, and all the blood had been carefully washed out of it, it was found to weigh nearly four pounds; the circumference at the base was eighteen inches; the length, seven and a half inches; the thickness of the wall of the left ventricle, one inch eight lines; that of the right, five or six lines; the right auricle, three lines, &c. In the duplicature of the lining membrane of its valves, there was found not merely fibrous tissue, but also some reddish muscular fibres, which were prolonged at the base of the valves into the fleshy substance of the heart: this was most obvious on the left side. The tendinous zones were very well developed, and at the base of the bicuspid valve there projected from beneath the lining membrane a sort of bony process, the base of which sank to the depth of an inch into the substance of the heart, widening out to the breadth of five or six lines as it descended. This, which M. Bouillaud seems to have mistaken for an accidental or morbid growth, is a natural structure, has been alluded to by Meckel and other comparative anatomists, and lately more fully described by Mr. Harrison, of Dublin.

The approximate results in respect to the mean weight and dimensions of the human heart, in the natural and morbid condition, which we have detailed above, though they are very important, and indicate great industry and zeal for science on the part of our author, are yet scarcely based on a sufficient number of individual instances to warrant our receiving them as settled standards of appeal; but it may fairly be expected that, when several observers, who possess an equally extensive field for observation, shall have instituted a similar series of experiments, and combined their results, such a standard, of some practical value to the morbid anatomist, may eventually be made out. In the mean time, even considered as approximations, they cannot fail to be valuable to the student of pathological anatomy; and we have therefore, at the risk of being considered tedious, given them in considerable detail.

The details will be best appreciated by those most inclined to pursue such enquiries.

When speaking of the morbid anatomy of the heart, M. Bouillaud very justly censures that slovenly method of dissection, (now, we trust, almost extinct,) which consists in merely cutting the heart across, and from such a section attempting to appreciate its several lesions; instead of slitting up its several cavities in regular succession, and carefully examining their absolute and relative capacities,—the condition and quantity of blood within them,—the thickness of their walls,—the state of their orifices, valves, tendons, fleshy pillars, and lining membrane,—of their vessels; in short, of every thing which enters into their structure.

In his chapter on the Motions of the Heart, one of the most important in the whole work, he commences by dividing them into the external or visible, and the internal or concealed, (valvular motions, &c.) In his historical view of the progress of opinions in respect to this branch of physiology, he goes back no farther than Haller: he alludes to Harvey but twice, we think, in the whole course of the work, and then in such a manner as not to impress us with the belief that he has ever read his works; both the passages cited by him being, if we mistake not, also given by Laennec, in whose book they might easily have been caught up at secondhand. We would not, however, quarrel with him on this account, were it not that Harvey's work not only was, but still is, one of the best on the subject of the heart's motions. Being written in the true spirit of the Baconian philosophy, and founded on experiment and observation, the conclusions detailed in it are in many points more correct than those which have been adopted by some physiologists of our own day: as, for instance, with regard to the coincidence of the contraction of the ventricles with the impulse against the chest, and the projection of the column of blood into the great arterial trunks, and also as to the precession of the auricular motion. He was, moreover, evidently aware of the fact which has been turned to so much account by Laennec, namely, that the motions of the heart are accompanied by distinctly audible sounds, "pulsam fieri et exaudiri in pectore contingit;" and compares the noise so produced to that heard in the throat of the horse while drinking. He combated successfully the opinion prevalent in his time, and supported by the great authority of Vesalius, and recently revived, that the beat of the heart was effected by the dilatation or diastole of the organ; as well as that its filling was the result of an active muscular expansion taking place in the walls of the ventricles.

Haller correctly described the contraction of the auricles as preceding that of the ventricles, the action of the latter being, in its turn, immediately succeeded by a period of repose. His view of this matter, which is certainly the true one, was departed from by Laennec, who conceived (probably on theoretical grounds, rather than from actual inspection of the action of the heart in a living animal,) that the auricular contraction followed that of the ventricle, intervening between the latter and the interval of repose; thus enabling him to account satisfactorily, as he thought, for the second or clear and short sound, as heard by the stethoscope. Mr. Turner brought us back to the older and true opinion, which has likewise been successfully advocated by Drs. Corrigan and Hope, and nearly all the latest and best writers on the subject; and is now adopted by M. Bouillaud, as being firmly and finally established. Whilst on this subject, our author alludes, in terms of merited approbation, to the labours of Dr. Hope, whose valuable work on the Heart he seems to have studied with much care.

The cause of the stroke of the heart against the side of the chest has lately given rise to numerous discussions. Dr. Corrigan and M. Pigéaux have ascribed it to the sudden swelling of the ventricles, on the injection of the blood into them in their diastole; a conclusion to which they were led, partly from observing that the arterial pulsations are posterior in point of time to the thoracic impulse, and partly from the difficulty of conceiving how the contraction of the ventricles could bring them in contact with the parietes of the chest. This opinion, which is, as we have seen, but the revival of an old and exploded hypothesis, has, as we observe by the minutes of the late meeting of the British Association at Dublin, been already abjured by one of its original advocates, influenced as well by his further investigation of the subject as by the arguments and experiments which have been brought to bear against it by various recent writers; and his relinquishment of it, and manly announcement of the same, display a candour and an openness to conviction which do him infinite credit.

The arterial pulse is no doubt felt distinctly subsequent, by a short interval, to the beat of the heart in parts at some little distance from this organ; but, as the space of time which intervenes between them decreases rapidly as we approach the heart, and near the origin of the great vessels is no longer observable, the fact does not really militate in any degree against the now almost universally received theory, which ascribes the impulse of the heart to the ventricular contraction. Indeed, the matter has been put to the test of direct experiment; for, in the report of a committee recently appointed by the British Association of Science to investigate the sounds and motions of the heart, published in the September number of the Dublin Journal of Medical Science, we find it stated that, "when the ventricles assumed their hardened state, their apex and a considerable portion of their anterior surface were closely applied to the sternum; and, when the hand was interposed between the latter and the surface of the ventricle, a strong compression was exercised on the fingers during each approach of the ventricles to the front of the chest." A similar observation was made by Dr. Hope in his experiments. It is mentioned, moreover, that when

"a small glass tube was introduced through a puncture in the right ventricle, a jet of dark-coloured blood was thrown forth during the globular and hardened state of the ventricles, and subsided when they became flattened and soft. A puncture was made in the pulmonary artery, close to the ventricle from which it arises, and through it a stream of blood issued synchronously with a jet from the tube in the right ventricle." From these experiments, taken conjointly, it appears to be incontrovertibly proved that the hardening of the ventricles is identical with their contraction or systole, with the beat of the heart, and with the arterial pulse in the commencement of the great vessels. The jet from the distant arteries,—the mesenteric or femoral, for example,—was observed to be posterior by an appreciable interval to that from the ventricle; a want of synchronism ascribable to the yielding and elastic nature of the walls of the arteries, which converts the direct or onward impulse of the column of blood into a wave-like or intermittently progressive motion. Were their walls, on the contrary, of rigid and unyielding materials, the mass of fluid, from its incompressibility, would be moved forward in totality, and the pulse would then really be synchronous all over the body, as it was supposed to be by Harvey and others; but, as it is, a portion of the onward impulse is expended, and time consumed, in dilating the arterial parietes, which again react, so as, by their alternate yielding and resiliency, to produce a pulsatile and wave-like motion.

According to M. Bouillaud, the stroke of the heart is effected by the apex alone; for, in three experiments made by him on the subject, he constantly observed that the point of the heart turned up towards the side of the chest during the systole of the ventricles, and that no real change of place of the entire organ occurred; and such is also the opinion of Dr. Williams of London, and of Dr. Hope. The experiment of the Dublin committee, detailed in a preceding paragraph, goes, on the contrary, to prove that the body of the ventricle, as well as its apex, is concerned in giving the impulse. In cases of morbid enlargement of the heart, but in them only, M. Bouillaud admits that the whole mass of the heart strikes against the thoracic parietes. In certain morbid conditions of the organ alluded to by our author in a subsequent part of his work, he conceives that the ventricular diastole likewise may sometimes cause a slight impulse, and even supposes that the contraction of the auricles may occasionally have a similar effect. He objects altogether to Senac's complicated explanation of the beat of the heart, according to which it was supposed to depend on the joint action of three causes,—namely, on the dilatation of the auricles at the same moment that the ventricles were contracting, together with the simultaneous dilatation of the aorta and pulmonary artery by the blood entering them; and also on a tendency in the arch of the former to straighten itself from the sudden impulse and direction of the fluid in relation to its curvature, (the tilting forwards of

Hunter.) To any one who has ever grasped the heart of a living animal in his hand, it must appear evident, we think, from the force with which his fingers are separated in its contraction, that the cause of the stroke resides in the ventricles themselves, and consists solely in their systole; at least, that this alone is quite adequate to its production. M. Filhos goes still farther, and asserts that the impulse depends on the left ventricle alone, by which the point of the heart is exclusively formed; and endeavours to account for it by the peculiar spiral arrangement of the muscular fibres near the apex of this ventricle; but to this view of the matter we cannot assent, both because the impulse to which we have just alluded is felt on both surfaces of the heart, and, as our own observation would lead us to think, is by no means confined to its apex, (and this is peculiarly obvious at the termination of a forcible expiration, more especially when the body is in a prone position, and turned a little on the left side; under which circumstances the whole mass of the organ seems to come into very extensive contact with the parietes of the chest;) and also because the shock of the heart may be very considerable in those cases of hypertrophy of the right ventricle where the cavity comes to form alone the entire apex of the organ.

As to the dilatation of the ventricles, it is ascribed, and we think correctly, partly to the elasticity of their fibres, and partly to the injecting powers of the auricles. The influence of the latter, however, is not rated very high by M. Bouillaud, who thinks that, had much depended on it, nature would have furnished the auricles with an ample valvular apparatus, against which to act, at the great venous orifices.

He next discusses the principle of the motion of the heart, and concludes, from the number of nervous branches transmitted into its structure, that this resides in the nervous system. Haller, as is well known, attributed it entirely to irritability, or a peculiar power inherent in the muscular fibres themselves. Bichat referred the origin of its motion to the ganglionic system; an opinion which was subsequently combated by Legallois, who, from numerous experiments, was led to place it in the spinal marrow: but there is every reason to think that his manner of viewing the subject was too exclusive, and that it is not on this portion of the nervous system that its motions immediately depend. Thus, M. Lallemand has observed that the heart beats in the fœtus, though wanting the spinal marrow; and Dr. Wilson Philip, Mr. Clift, and M. Breschet, have seen the pulsations of the heart continue after the destruction of this portion of the nervous system alluded to, and more especially when young animals were the object of experiments, and when the injury was inflicted slowly. M. Breschet thinks he has established the opinion of Willis and Bichat by certain experiments, in which the heart's action was immediately cut short by the section of the cardiac plexus on removal of the cardiac ganglion, and looks upon it as being now a settled point that the primary cause of the vital action of the heart resides in the ganglionary system. M. Magendie, however, has been less fortunate in similar experiments instituted with the same view; for the violence of the operation necessary for the extraction of the cervical ganglions and of the first thoracic one, was so immediately followed by death, that he could make no satisfactory deductions as to the influence exerted thereby on the heart.

M. Humboldt found that the muscular contractions of the heart removed from the chest were rendered much more frequent and forcible by means of galvanism, applied to one of the cardiac nerves laid bare by dissection; and Home and Weinhold observed a similar result from irritating the great sympathetic, and establishing the galvanic current through the medium of one of the thoracic ganglia, detached and turned up on the heart. That injury of the spinal marrow has, notwithstanding all this, great influence on the heart's action, is admitted in the work before us, but ascribed to the sympathy of that portion of the nervous system with the ganglia of the sympathetic. The story of Colonel Townsend, who is said to have had the power of voluntarily stopping the action of his heart, is doubted by M. Bouillaud, but we think without sufficient cause.

In his elaborate analysis of the sounds of the heart and their causes, our author enumerates four conditions, one or other of which may be conceived as being concerned in their production: viz. the impulse of the heart against the side of the chest; its friction against the pericardium; the passage of blood through its cavities; and the alternate rising or falling of its valves: and to these we would add a fifth, the sound of muscular contraction (*bruit musculaire,*) which in a muscle, the contractions of which are so energetic, may be considered capable of being very audible. Before entering into this part of his subject in detail, he meets the point as to whether it be possible to distinguish clearly from one another the sounds caused by the action of the right side of the heart from those of the left, and concludes with Laennec in the negative, as far as the normal condition of the organ is concerned; whilst he freely admits, at the same time, that, in certain diseased states, we may determine not only to which side of the heart the anormal sound belongs, but even in which of the orifices, the arterial or auriculo-ventricular, it takes place. If we endeavour to ascertain precisely the seat of each of the two sounds, the long dull sound and the short clear one, we shall find, says he, that the first has its maximum of intensity immediately below and rather to the outside of the nipple, that is, in the point corresponding to the mitral valve; and that the maximum of intensity of the second sound exists immediately above and to the inner side of the nipple, or in the point corresponding to the sigmoid valves.

M. Laennec has greatly under-rated the extent of surface over which the sound of the heart is audible in the normal condition: thus, even in thin narrow-chested subjects and young children, in whom it is the most extensively heard, he limits it to the sternum, the anterior and upper part of the chest on the left side as high as the clavicle, and less sensibly over the same portion of the right side; whereas M. Bouillaud has, many hundred times in such subjects, heard it not only in the situations above mentioned, but also in all the other regions of the chest, not excepting even the right posterior regions; as also in the lateral portions of the neck, and here sometimes almost as loudly as in the præcordial region. These sounds become augmented or diminished, not merely in peculiar morbid states of the heart itself, but are also, as pointed out by Laennec and subsequent writers, capable of being modified both as to their situation and intensity by the condition of neighbouring organs, independently of any disease in the central organ of the circulation. For example: the sounds are transmitted in a much more intense form through a hepatized lung, or one condensed by the pressure of a fluid in the pleural sac; through tubercular masses or tumours, or through the indurated walls of a tubercular cavity; and the place where the beat of the heart is felt and its sounds are heard may be quite changed by the presence of a large pleuritic effusion.

As to what is the true theory or explanation of the sounds of the heart, M. Bouillaud admits that, notwithstanding the numerous discussions which have taken place on the subject, the majority of physiologists are still undecided. He hopes, however, in the present work, if not to have finally settled the question to the satisfaction of all, at least to have succeeded in removing a portion of the obscurity with which it was enveloped. He commences by giving a rapid sketch of the various opinions which have been brought forward on the subject. 1. M. Laennec, though he abstained from offering any thing like an explanation of the matter, so far as the normal sounds are concerned, yet, seeming to anticipate that they would by others be attributed to the concussion of the heart against the thorax, insists on the fact which, if universally true, is hostile to this view, namely, that the sound is loudest when the ventricles are thinnest and the impulse consequently feeblest. 2. M. Pigéaux ascribes the sounds to the friction of the blood against the sides of the cavities in the one case; and against the walls of the great arterial trunks on the other: the first producing the long dull sound, which he erroneously conceives to be coincident with the ventricular dilatation; the second, the sharp clear sound, which, with similar incorrectness, he supposes to be synchronous with the ventricular contraction. Views of the same kind were also advocated by Drs. Corrigan, Stokes, and Hart. 3. M. Marc D'Espine asserts that both the sounds are the simple effect of the systole and diastole of the ventricles, and quite independent of the play of the valves, the friction of the blood, &c. 4. M. Rouanet attributes both the sounds to valvular action; the first to the sudden sonorous distention and mutual collision of the auriculo-ventricular valves during the systole of the ventricles; and the second, or clear sound, to the reaction of the column of blood against the sigmoid valves: and this is the theory, too, which, with a few slight modifications, the author of the work before us adopts. The sudden extension of any thin membrane or artificial tissue gives rise, as M. Marc d'Espine remarks, to an audible sound, clear in proportion to the tenuity and unyielding nature of the texture; and in both these respects the cardiac valves are well qualified to produce sonorous vibrations.

To prove that the second sound may depend on the falling back of the blood against the sigmoid valve, he adduces the following direct experiment:—He fixed the end of a short glass tube, of an inch bore, into that portion of the aorta which is immediately below the sigmoid valves, and attached the other end to a bladder full of water. Into that part of the artery which lies above these valves another tube, of the length of four feet, was fixed: it being made so long in order that the weight of the column of fluid should be sufficient fully to compensate for the reacting force existing in the natural state. The portion of the artery corresponding to the valves was then seized in one hand, and held close to the ear, whilst with the other hand the bladder was suddenly compressed at intervals, so as to jerk up the fluid, and thus in some degree to imitate the heart's projective power. Each time that the pressure on the bladder ceased, and the column of liquid was allowed to fall back on the valves, a sound very analogous to the second sound of the heart was heard; as much so, in short, as could be expected under the great dissimilarity of circumstances,—the absence of the resonance of the thorax, the want of the vital reaction of the tissue, and of the compression of neighbouring organs. Dr. Corrigan, however, states his having recently made a somewhat similar experiment with a very different result. He cut out from the heart of an ass the ascending aorta, with its valves, and "tied it on the end of a leaden tube, of a corresponding diameter, and about five feet long. About two or three inches of the aorta then being free from the lower extremity of the tube, in this state, holding the sides of the aorta together below, he filled the tube with water, and then, placing the thumb on the upper end, so as to close it, the fingers were withdrawn from the lower end, and the upper end still remaining closed, the external pressure of atmospheric air kept the two sides of the aorta below together, and no fluid escaped. The ear was then applied to the lower end of the tube, close to the aorta, and, the thumb being suddenly withdrawn from above, the whole column of fluid came suddenly down, and distended the aorta and valves; and yet there was no sound whatever similar to the second produced. He attached to the end of the leaden tube a piece of sounding-board, to assist the ear, and the result was the same as before."

It is difficult to reconcile the very different results of these two experiments. The arrangement in the experiment of M. Rouanet obviously approached much nearer to the natural state of things than in that of Dr. Corrigan, where the commencement of the aorta, in place of being constantly in a tubular and more or less distended state, as it always is during life, was in a collapsed condition, with its opposite sides in close contact, from the effect of atmospheric pressure. The throwing back of the valves in the latter case would take place under totally dissimilar circumstances to what it does in the natural state.

5. Dr. Hope attributes the first sound (we may now say *attributed*, but his late Appendix had not been seen by M. Bouillaud when the present work was published,) to the collision of the molecules of the blood amongst themselves; numerous and diversified currents being produced therein by the irregularity of the internal surface of the ventricles, columnæ carneæ, &c.;—and the short clear sound to the reaction of the walls of the ventricles on the blood thrown into them by the auricles; the fluid receiving a sudden check on the former cavities reaching their extreme limit of extension.

6. M. Magendie ascribes these sounds to the double impulse of the heart against the walls of the thorax: the first, or dull sound, being caused by the stroke of the apex in the systole; the second, or short clear one, by the body of the heart coming into forcible contact with the sternum in the diastole; its superior clearness being attributed by him to the greater sonoreity of the sternum, as compared with the cartilages of the ribs. He asserts, moreover, that the heart's action produces no sound if the sternum and ribs be removed, and its external impulse thus prevented: but here he is certainly in error, as we shall afterwards see.

7. M. Piorry has advanced the notion, at variance with the observation of nearly every one who has occupied himself on the subject, that the ventricles do not contract simultaneously, and that the dull sound is produced by the left ventricle, and the clear one by the right. For any one who wishes to convince himself of the incorrectness of this hypothesis, it is only necessary to introduce a small tube into each of the ventricles of a living animal, or into the pulmonary artery and aorta, close to their origin, and observe the perfect synchronousness of the jets from each; or, what is still simpler, merely to grasp the heart in the hand, and feel (as we have often done) the simultaneous hardening of the two ventricles in their systole.

8. Mr. Carlisle has endeavoured to account for the first sound by the projection of the blood against the walls of the great vessels, and for the second by the reaction of the fluid against the sigmoid valves. But, as the first sound persists after the great vessels have been cut across, the first portion of his hypothesis appears untenable, and has, we believe, been relinquished by its author.

To the above theories we may add that of Dr. Williams, one of the most indefatigable and cautious investigators of the subject, who ascribes the first sound to the muscular contraction of the ventricles (*bruit musculaire*), and the second to the reaction of the arterial column against the semilunar valves.

All the above hypotheses, when analyzed, are reducible to four elements:

1st. Muscular action. (M. d'Espine, both sounds; Williams, and the Dublin Heart Committee, first sound.)

2d. Friction of the blood against the walls of the containing cavities or vessels, (Pigéaux, Carlisle;) and consequent currents, (Hope.)

3d. Double impulse of the heart against the side of the chest. (Magendie, &c.)

4th. The play of the valves. (Rouanet and Bouillaud, both sounds; Williams, Carlisle, and the Dublin Heart Committee, second sound.)

It is rather by inference than in consequence of any direct assertion on his part, that Laennec has been sometimes classed amongst those who ascribe the sounds in question to the muscular contraction, (*bruit musculaire.*) It was more particularly with a fancied similitude between the *bruit musculaire*, as heard in the voluntary muscles, and the *bruit de soufflet*, or bellows sound, that Laennec appears to have been struck: and, from the fact of his having instituted such a comparison, M. Bouillaud is of opinion that he must have mistaken the sound elicited by the *friction* of the clenched fist applied against the ear (the mode in which he investigated the sound produced by a muscle in action,) from that really resulting from a vibratory motion in the muscular fibres themselves. The distinguished pathologist above named has also, he conceives, fallen into a manifest inconsistency, in at once referring the bellows sound to the muscular action of the ventricles, and at the same time asserting that it is of more frequent occurrence during their diastole, or state of relaxation, than during their systole. In point of fact, it may take place either during the systole or the diastole, or both; but it is a much more frequent accompaniment of the first. M. Bouillaud does not recognize the *bruit musculaire* as a cause either of the natural sounds of the heart or of the bellows sound. He does not deny its existence, but considers it of quite too feeble a character to account in any degree for the cardiac sounds.

To the theories which ascribe the natural sounds of the heart to the impulse of the blood against the walls of its cavities, to friction, to currents, &c., he is equally hostile. That this fluid, in its passage through the several orifices, may produce a slight sound, even in the normal condition, and very marked ones in certain morbid states, he is very far from denying; but he asserts that such are, under all circumstances, very unlike the natural *tic-tac* sound of the heart. If such were really the origin of the normal sounds, they should not altogether disappear, as they are known to do, in certain diseased conditions of the valves, and become entirely supplanted by those new or accidental sounds known under the name of bellows sound, rasp sound, &c. Any sound which may be thus produced in the natural state is, he asserts, entirely eclipsed by the valvular sound afterwards to be enlarged upon.

In refutation of M. Magendie's hypothesis, our author adduces the following experiments, lately made by him. He laid bare the heart of a vigorous cock, and applied alternately the stethoscope and the naked ear to it, both whilst still within the pericardium, and after the removal of that covering; and constantly heard the double sound of the heart quite distinctly, though no contact was permitted between it and the thoracic parietes: by way of precaution, he even raised it on his finger out of the chest. On the interposition of a cloth between the heart and his ear, to protect the latter from the contact of the blood escaping from the wound, it was still audible, though less clearly so. When the stethoscope was employed, the friction of the heart against its end produced a peculiar noise; but this was single, and of a rubbing character, which it was quite impossible to confound with the natural sound of the heart. The same experiment has also been made by Dr. Hope and Dr. Williams, in London, and the committee above alluded to in Dublin, on larger animals, with precisely similar results. By the last-mentioned experimenters it is even stated that the sounds could be heard, though feebly, without bringing the ear into absolute contact with the organ. These facts seem totally incompatible with the theory which ascribes the sounds to external impulse. On cutting the great vessels across, and removing the heart from the chest, the pulsations continued for some instants; but in this, its empty state, M. Bouillaud could hear no sound. Here, however, his observations are at variance with those of the authorities just alluded to. Had his experiment been made on the heart of a larger animal, as an ass or a calf, for example, the organ being removed from the body whilst still in a state of vigorous action, we have no doubt he would have succeeded in hearing at least one of the sounds (viz. the first,) very satisfactorily. Dr. Hope's experiments, which were the first of the kind, were quite unknown to our author at the time of his instituting his own.

Of M. Rouanet's, or the valvular theory, M. Bouillaud is, as already stated, a warm advocate. He does not pretend to have produced absolutely direct proof of the sounds depending on the play of the valves; but having, as he fancies, negatived all the other hypotheses advanced, no other possible explanation than this is, he thinks, left us. Pathology also seems to him to be highly favorable to this view; for it is when the valves are deeply altered in their structure, or in their relation to the orifices, and rendered thus unfit for their office, that the sounds of the heart come to deviate most from their natural condition; and in proof of this he refers triumphantly to the cases throughout his work. The chief cause of the first sound, according to M. Bouillaud, (and the only one according to M. Rouanet,) consists in the sudden elevation and collision of the opposed surfaces of the auriculo-ventricular valves, and especially of those of the left side: in addition to which, he conceives that the rapid throwing down of the sigmoid valves against the arterial walls by the projected column of blood may also assist in its production. If any sound be really produced by the friction of the blood against the orifices, its character is quite lost, as already stated, in the valvular noise.

With regard to the second sound, he coincides with Dr. Carswell, who first suggested the idea, and with M. Rouanet, who subsequently developed it, in believing that it originates in the elevation of the sigmoid valves, which is effected partly by the reaction of the arterial tubes on the column of blood in them, and partly by the suction of the ventricles in their diastole. He is also disposed to ascribe somewhat to the sudden falling back of the auriculo-ventricular valves, (under the influence of the cause just mentioned, as well as in consequence of the rush of blood over them from the auricles.) We confess, however, that, in regard to his explanation of the first sound, we see an insuperable objection in its prolonged and uniform character, which seems quite inexplicable by the rapid and momentary motion of the auriculo-ventricular valves.

M. Bouillaud was not aware, apparently, of the direct experiments performed in the spring of last year, by which Drs. Hope and Williams have endeavoured to decide how far the valvular motions are concerned in the production of the sounds. According to these, it seems that the first sound persists even after the action of the auriculo-ventricular valves has been altogether impeded by thrusting the auricles through the corresponding orifices into the cavities of the ventricles; but that the second sound is put an end to, and succeeded by a hissing noise, on transfixing one or more of the sigmoid valves of the aorta and pulmonary artery with a curved awl or needle, and thus confining them against the walls of the vessel, and preventing their action; a result which has been very recently verified by the repetition of a similar experiment in Dublin. The second sound may likewise be stopped by compressing the aorta and pulmonary artery, and so preventing the falling back of the column of blood against the sigmoid valves; but this experiment is less satisfactory than the preceding, as the pressure requisite generally gives rise to a considerable degree of disturbance in the heart's action. The first sound is found to be loudest over the middle of the ventricles; the second, over the sigmoid valves, and for a few inches upwards. The conclusion at which Dr. Hope arrived from the experiments above alluded to, and which he has published in his recent Appendix, differ very much from those brought forward by him in 1832. His present idea of the first sound is that it is of a compound nature, consisting, "1st, possibly of a degree of valvular sound; 2d, of a loud smart sound, produced by the abstract act of sudden jerking extension of the muscular walls, which, to avoid circumlocution, he calls the *sound of extension*;" "3d, a prolongation, and possibly an augmentation of this sound, by the sonorous vibrations peculiar to muscular fibre, (*bruit musculaire;*)" but he thinks, considering the extraordinary intensity of the sound of the heart during palpitations, that it would do violence to all analogy to suppose it produced solely by the *bruit musculaire.* He seems still, moreover, inclined to attribute to the motions of the blood itself some share in its production. His account of what he calls the sound of extension, which, as we have just seen, he considers to be the chief cause of the first sound, does not appear very precise, or indeed altogether intelligible. With regard to the second sound, also, he has completely renounced his old opinion of its depending on the collision of the blood in the diastole of the ventricles, and adopted that of M. Rouanet, Mr. Carlisle, &c., who ascribe it to the reaction of the blood against the sigmoid valves. Dr. Williams's conclusions, from the very same set of experiments, are, so far as the cause of the first sound is concerned, quite different from those of Dr. Hope: he adheres, in respect to it, to his original opinion, published in 1828, and in the advocacy of which he at first stood alone, namely, that it depends on the *bruit musculaire.* He remarks, in regard to Dr. Hope's objections to this cause, that "it is very possible to imitate the first sound of the heart by suddenly contracting the muscles of the gently closed hand; but it is not to be expected that the sound of muscles *remaining* in a state of tension can give a similar sound. These produce the dull rumbling sound described by Dr. Wollaston, which Dr. Hope seems to have taken as the type of muscular sound. The muscular contraction of the heart is preeminently calculated to produce sound, inasmuch as the motion is strong, abrupt, and simple, and the resistance considerable, and continued as long as the semilunar valves are open and the auricular shut." He shews very satisfactorily that it cannot depend on the action of the auriculo-ventricular valves, (for it continued after they were partially destroyed, or their motions obstructed;) nor yet on the collision of the particles of blood, (for it is heard even after the heart is empty, and, we may add, even after its removal from the body. All Dr. Hope's three causes, he thinks, are included in the simple principle of the sudden tightening of muscular contraction. With regard to the second sound, he was formerly inclined to Dr. Hope's original opinion; but his recent experiments on the subject have induced him to adopt the hypothesis which ascribes it to the sigmoid valves. It is obvious, amidst all this diversity of opinion, that there is still ample room for further investigation of the sounds of the heart; and we observe with pleasure, that Drs. Todd, Williams, and Clendinning have been appointed by the British Association to prosecute the subject; and to the result of their labours, as well as of those of the Edinburgh committee, we look forward with much interest.

M. Bouillaud next proceeds to investigate the alterations in the rhythm of the heart induced by disease, as also in the number and force of its pulsations,—the extent in which they can be felt, together with their morbid or accidental sounds, as well as those occasionally heard in the arteries, and those which accompany pregnancy.

The duration of the systole, says our author, seems often to be prolonged by the difficulty experienced by the blood in escaping through the morbidly altered arterial orifices. Now, this appears to us to be a great difficulty in the way of the exclusive valvular theory adopted by him. The prolongation of the first sound under such circumstances is just what we should expect, if this sound depended on muscular contraction; whilst, on the other hand, we cannot see how the valvular sound could be so drawn out, being in its very nature, we conceive, brief, or almost instantaneous. In some rare cases, he has, in addition to the shock caused by the ventricular systole, felt a second, or even a third impulse, which, as already stated, he ascribes to dilatation of the ventricles; though we apprehend that the phenomenon might more satisfactorily be explained by a repetition of abortive or spasmodic contractions, unaccompanied by arterial pulsations, in consequence of the ventricle being in a nearly empty condition. We have mentioned that he also believes (and the opinion is not peculiar to him,) that the systole of the auricles is capable, in certain morbid states, of causing a notable impulse. Thus, in a woman labouring under an enormous hypertrophy of the heart, with indurations of the mitral valves, there was a very obvious pulsating motion in the left superior mammary region, about an inch below the clavicle; and the finger applied here was thrown up with a well-marked shock, which, as the impulse of the ventricles was felt two inches lower down, could be accounted for by nothing else than the systole of the left auricle, in a state of hypertrophy and dilatation. It alternated with another motion, which seemed coincident with and dependent on the auricular diastole; and this double or undulatory motion of contraction and dilatation, as felt through the emaciated and distended parietes, imitated perfectly that seen in the auricles of the naked heart. Long-continued and violent palpitations, particularly in cases of hypertrophy, tend eventually to produce a very marked prominence in the præcordial region. This, which he has often had occasion to observe, has not previously, as far as he knows, been noticed by any other writer; though, from our knowledge of what occurs in the case of aneurismal tumours, it is what we might *a priori* have expected.

With regard to the vibratory sensations imparted to the hand applied over the cardiac region, (*frémissement cataire* of Laennec,) which was first pointed out by Corvisart as diagnostic of narrowing of the left orifices, a very accurate idea may be obtained by touching a sonorous body in vibration,—the larynx in speaking, or an aneurismal varix. It is singular that Corvisart, who was aware of a similar thrilling sensation imparted to the finger in certain cases of disease, which he erroneously supposed always to consist in contraction of the aortic orifice, should not have been apprized of its still more frequent presence in the larger arteries, in which it sometimes coexists with, and in others is altogether independent of, disease in the heart; and which has since been so well described by Laennec. It exists in nearly all those singular cases where pulsations of the arteries are accompanied by audible sounds, of which that designated by Bouillaud under the strange name of "*bruit, ou ronflement du diable,*" is one of the most remarkable. He believes that both this and the bellows sound (with which it is so closely connected) depend on narrowing of one of the orifices of the heart, and the consequent increase of friction of the current of blood against the edges of such diseased orifices. Laennec, on the contrary, though aware of the frequent coexistence of the *frémissement cataire* with the above lesion, yet seems to have looked upon it as having its immediate source in a spasmodic state, rather than in this peculiar organic change, inasmuch as he had frequently found it of equal intensity in the absence of the latter; and even M. Bouillaud admits that there exist numerous instances of it in which there is no such narrowing of these orifices, and that we are as yet far from having attained to an adequate knowledge of all the possible causes of this phenomenon. Another of these causes consists in the friction of the opposed surfaces of the pericardium, thickened and rendered rough by effused lymph; and here the vibratory thrilling sensation coexists with the *bruit de rape*, or sometimes with that sound compared by Colin to the creaking of new leather. In one instance given in the present work, it was connected with the friction caused by a rough, irregular ossification on the surface of the heart. He suggests the possibility of its occasionally depending also on the reflux of blood into the ventricles or auricles.

As to the *anormal* sounds of the heart, it was to have been expected that Laennec should have fallen into some errors, being in great part ignorant of the true causes of the double sound in the natural state. The passage of the blood through the several orifices of the heart may, as it has already been admitted, occasionally be productive of some slight sound. Now, though this, in the healthy condition, is drowned in the valvular sound, yet, in proportion as the orifices become narrowed and irregular in their shape, and the valves diseased, this frictional sound will become not only altered in character, but so increased in intensity as eventually to mask the normal sound, which has become changed and diminished under the influence of the same causes.

The sounds of the heart vary greatly in intensity in different individuals, being in some instances as feeble and as difficultly heard as in the fœtus at the fifth month; whilst in other cases it has been heard at the distance of two or more feet from the chest. The loudness depends, according to our author, not on the comparative thinness and capacity of the ventricles, as M. Laennec conceived, but on the tenuity of the valves, the energy with which they are thrown into action, and the tenseness they are capable of assuming. No doubt the increased thinness of the ventricles, and that of the valves, frequently coexist; as does hypertrophy of the former with thickening of the latter; and hence perhaps the error of Laennec. In the case of hypertrophy, not only is the state of the valves unfavorable to the production of sound, but also the thick mass of muscle in the walls of the heart so affected, is very ill suited for conducting it.

Of the principal morbid varieties in the sounds of the heart, one is that where they assume a hard dry character, as if the valves were formed of parchment; and this generally depends, as dissection proves, on a state of considerable hypertrophy and rigidity of the valves of the left side, and especially of the mitral valve. Whilst those cases where it takes on a hoarse, large, rough character are usually connected with a fungoid and infiltrated condition of the valves, which are then soft and flaccid, instead of being firm and resisting, as in cases of true hypertrophy. When this species degenerates into a bellows murmur, it is from incrustations or vegetations being developed in the valves. The natural valvular sounds, as he calls them, never, according to his observation, disappear entirely in any case where the lesions of the valves are uncomplicated, and have not yet gone to such an extent as to prevent their proper motions. But if there exist at the same time narrowing of an orifice, or roughness and irregularity of the surface of the valves, or if they are no longer capable of fully closing the orifice and preventing the reflux of blood, the natural sound becomes mixed with, and modified by the bellows murmur, the saw or rasp sound, &c.

In addition to the various anormal sounds of the heart hitherto mentioned by authors, M. Bouillaud has noticed a new one, analogous to the *bruit musical* of the arteries described by Laennec; and he compares it to the cooing of the turtle dove, or sometimes to the chirping of small birds, or to the *râle sibilante* of bronchitis. Of this he has as yet met with but two cases, and M. Rouanet with a third: they seemed to be connected with narrowing of the orifices.

The saw sound has in some cases a peculiar hissing character, which may be accurately imitated by a long drawing out of the letter S; whilst in others it has a more thick and rough tone, of which a pretty accurate notion may be imparted to those who have not yet heard it, by pronouncing the letter R with a prolonged burr. All of these sounds, which may be considered as so many modifications of the *bruit de soufflet,* seem to M. Bouillaud to be very commonly connected with narrowing of the orifices of the heart. Laennec, on the contrary, looked upon them as the result, not of organic lesion, but of simple spasm in the heart or arteries.

The effect of narrowing of the passages through which the blood flows, in producing the bellows sound, may, as is well known, be artificially produced in the arteries by making a certain degree of pressure on them with the stethoscope. The friction of the current is thus increased, and this peculiar sound ensues. M. Bouillaud asserts, that, out of upwards perhaps of a hundred cases, where contraction of the orifices with induration of the valves was established by dissection, the bellows sound had been observed during life in all, with a single exception, and that had not been very carefully examined.

M. Piorry's experience is, however, much at variance with this: neither the *bruit de soufflet* nor any of its modifications were present in nineteen twentieths of such cases of this kind as had fallen under his own observation. But here, as in other instances, where negative proof is set in opposition to positive, we feel inclined, *cæteris paribus,* to give most weight to the latter, until additional and more extensive investigations shall have finally settled the point. Even Laennec himself admitted the very frequent coincidence of *bruit de soufflet* with narrowing of the orifices, though he did not consider the former a direct physical consequence of the latter. That it is an absolutely necessary consequence of such a condition of parts, we cannot ourselves admit; as we have reason to think that cases of well-marked contraction, with ossifications, &c., do occasionally present themselves, unaccompanied by any such abnormal sounds. Such occurrences are however, we believe, extremely rare, and form only the exception, and not the rule, as M. Piorry would have them to do. In forming our diagnosis, it should never be forgotten that the peculiar sounds in question may be produced by the refluent as well as by the onward motion of the blood; a circumstance which has lately been enlarged upon at some length by M. Filhos, in France.

Though M. Bouillaud looks upon the narrowing of the orifices with induration of the valves as a very frequent cause of soufflet, he does not by any means maintain that it is the only one, but recognizes several others, amongst which the following are the principal: 1. Polypous concretions formed during life; and such he thinks is probably one of the causes of the bellows sound in acute inflammations of the internal lining membrane of the heart. 2. Smallness of the aortic orifice, even when the valves are perfectly healthy. This condition, whether congenital or acquired, coexists very often with dilatation and hypertrophy of the left ventricle; circumstances which will necessarily add much to the intensity of the sound. 3. Vegetations and calcareous incrustations on the valves, with irregularity of their surface, even when their efficiency has not been altogether destroyed thereby, nor the size of the orifices manifestly affected. The infiltration of the valves from endocarditis he conceives also to be a frequent cause of the sound. 4. Preternatural adhesions of the auriculo-ventricular valves to the adjacent parietes of the heart. 5. Dilatations of one or more of the orifices of the heart, with consequent inefficiency of the viscus. 6. Hypertrophy, with dilatation of the left ventricles, even though unattended by narrowing of the orifices, is occasionally accompanied by the bellows sound; but here it is generally intermittent, and only well marked at such times as the motions of the heart have been unusually accelerated. 7. He has in some very rare instances met with it in the heart of chloretic, nervous, anæmic individuals, and that especially during the presence of palpitations. 8. Great and sudden debility from hemorrhage, and other lowering causes, gives rise to the temporary appearance of this phænomenon in conjunction with an extremely rapid and feeble pulse. Dr. Hope, in experiments performed in conjunction with Dr. M. Hall, succeeded in producing it at pleasure in the lower animals, by repeated abstractions of blood. It is suggested by M. Bouillaud that it may depend on a narrowing of the orifices, which thus endeavour to adapt themselves to the diminished quantity of fluid circulating through them. A spasmodic action in the heart (for that spasm is an ordinary consequence of extreme depletion, in the voluntary muscles at least, is incontestable,) should, we think, also be taken into the account.

All the above cases, according to our author, are reducible to one common principle, viz. increased friction, produced in some of them by the direct, in others by the refluent, current of the blood. If this be correct, it seems to follow that, from *bruit de soufflet* alone, it must be quite impossible to decide in which of the orifices, if any of them, the disease is seated; for, though it be coincident with the ventricular contraction, it may depend either on the rush of blood through a diseased aortic or pulmonary orifice; or on a reflux through the auriculo-ventricular openings, or both; and if, on the other hand, it coexists with the diastole of the ventricle, it may originate either in reflux through the former openings or depend on a morbid alteration of the latter, or on both circumstances together. In fine, *bruit de soufflet*, properly so called, may exist with, or without, organic change in the heart. When it depends on contraction of the orifices, or induration of the valves, the changes of structure are generally rather of a fibro-cartilaginous than of an osseous nature. When the latter species of organization exists, it seems to give rise to those rougher varieties of abnormal sounds known by the name of *bruit de rape*, *bruit de scie*, &c. The sibilant or musical sound M. Bouillaud believes to be always connected with narrowing of the orifices, or a diseased state of the valves, it being merely a modification of the bellows sound in its highest degree of intensity.

Of those cases, which M. Bouillaud was the first to recognize, in which, in place of the double sound of the heart, we have three or even four sounds, he gives a short account of three examples, and alludes to several others. The first was in a woman where there were four successive sounds during each revolution or complete beat of the heart. The first sound was composed of a mixture of the normal first sound with a slight bellows sound; two sounds of a dry hard character (*craquement sec*) followed in rapid succession; and then came the fourth, which was a pure bellows sound. This case was examined not merely by M. Bouillaud, but also by several of the other physicians of La Charité and a number of the pupils, by all of whom the existence of the above phenomenon was confirmed. On dissection, there was discovered very considerable narrowing of the left auriculo-ventricular orifice; a fibro-cartilaginous state of the mitral valve, tendinous thickening of the aortic valves at their adherent edge; and fibro-cartilagious patches, and some adhesions of the pericardium. The second case was that of a young woman labouring under a very acute rheumatic inflammation of the inner lining of the heart, which was followed by all the symptoms of valvular induration and contraction of the left auriculo-ventricular opening. A triple sound was heard in this individual, which he compares to the drums beating the "rappel," or to the noise produced by a hammer let fall on an anvil, and rebounding twice, (tīc...tăc... tăc.) The first of these was accompanied by a slight bellows murmur; the two others followed in rapid succession, and seemed to be but a division of the second sound, as if the ventricle had not been able to fill itself completely on the first effort at dilatation. The same case, a few days after, presented a fourth sound in the form of a terminal soufflet succeeded by a short silence, and then a repetition of the whole. The third case given was that of a young man where the triple sound was heard: the pulse was dicrotous, as if the ventricle was unable to empty itself at a single contraction. The second arterial wave seemed to be extremely small. He believes that, in all such cases as those just detailed, there exists a narrowing of some one of the orifices of the heart and induration of the valves. There is an opposite class of cases, in which the natural double sound of the heart is as it were absorbed by a single but very loud bellows sound; and here the second sound seems, as it were, absent, the two bellows sounds being run into one. This seems to be owing in part to the rapidity of the pulse usually observed in such cases; and he mentions an example where the two sounds, so confounded, became separate on the pulse being reduced by the action of digitalis. Sometimes, by merely removing the stethoscope to a point at some distance from that where this *bruit de soufflet* is at its maximum of intensity, the distinction into two sounds becomes sufficiently evident. He believes, however, that the second sound may not only cease to be distinguishable, but be really wanting, namely, in cases of extreme debility.

Though he disbelieves in the production of any sound by the heart's impulse in the natural state, yet it seems to him not only possible but certain that, when the heart's action becomes very violent, the case is otherwise; the shock against the side of the thorax being then productive of a kind of clear metallic ringing sound, as already observed by Laennec; possibly also in cases of ossification of the heart or pericardium, the same phenomenon may occur; though he admits that he is not as yet in possession of any facts illustrative of this latter cause. This metallic sound may be imitated very exactly by applying one hand over the ear, and tapping on the back of it with a finger of the other. It is a sound which in no wise interferes with, and is incapable of being mistaken for, the double sound of the heart, and is heard only during the systole.

The abnormal sounds occasionally produced by the friction of the opposed surfaces of the pericardium, when in a diseased state, seem to have escaped the observation of Laennec. They are divided by M. Bouillaud into three species: 1. The rubbing sound, ("*bruit de frottement,*") which very closely resembles that heard in certain cases of emphysema of the lung, and may be pretty closely imitated by rubbing together two pieces of silk, bits of parchment, or bank-note paper. It may be distinguished from a similar sound taking place on the surface of the lungs, by its being double, but synchronous with the heart's motion. It is most obvious in the systole, and is diffused over a considerable surface; a circumstance which may aid us in discriminating it from the morbid frictional sounds accompanying certain lesions of the valvular orifices above alluded to. 2. The creaking sound, or that which by M. Colin, who first observed it, was compared to the sound of new leather, as heard in the creaking of the sole of the shoe in walking, is of infinitely rarer occurrence than the preceding, having been met with but once by our author in a state of purity, and by Andral likewise but once. 3. A scraping sound, ("*bruit de râdement,*") such as might *à priori* be expected to be produced by the rubbing a hard and rough cartilaginous or osseous body against the pericardium; a very remarkable instance of which is recorded in the work before us: the ossific deposit having commenced in the mitral valve, extended through the whole thickness of the left ventricle, raised the external membrane of the heart, and projected into the cavity of the pericardium precisely opposite to the point where the scraping sound had been heard during life. Its synchronism with the motions of the heart will enable us to distinguish it from somewhat similar morbid sounds which have their origin in the cavity of the pleura or in the bronchi.

The first two of the sounds above mentioned have hitherto been only met with in acute pericarditis. The faintest kind of frictional sound he believes to take place only in the incipient stage of this disease, while the membrane is preternaturally dry, and before effusion of any kind has commenced. The diffused rasp sound and saw sound he believes to indicate the formation of these thick, rough, areolated false membranes, found in the more advanced stages of the same disease; and the creaking, leathery sound he thinks takes place when the false membranes are peculiarly dense, firm, and elastic, and have perhaps already given rise to adhesions which are subjected to incessant dragging in the motions of the organ: such at least was the state of things in the single case alluded to above.

As to the bellows sound which so frequently accompanies pericarditis, he believes that it depends on the coexistence of an endocarditis, or inflammation of the internal membrane of the heart, and the consequent affection of the valves, the production of polypus concretions, false membranes, &c. within the heart, and not on the mere increase of action in the ventricles, as some writers suppose. Dr. Latham, who first observed the coexistence of *bruit de soufflet* with pericarditis, erroneously conceived that it was peculiar to the rheumatic variety. Dr. Hope corrected this too narrow view of it, and has, along with Dr. Stokes and others, recorded additional examples of its existence; but the first two abnormal sounds alluded to above, though much more characteristic of this disease than the bellows murmur, seem to him to have been very generally overlooked: not, however, by Dr. Stokes, (though our author has neglected to except him,) by whom a very valuable paper on pericarditis was published in the autumn of 1833, in which both the sound of "*frottement*," and that compared to the creaking of new leather, are particularly insisted on; and Mr. Mayne, still more recently, has added a new value to these signs by stating more precisely than had hitherto been done, the period of the disease at which they are ordinarily to be found.

M. Bouillaud does not think it has ever yet been demonstrated that a simple pericarditis can give rise to *bruit de soufflet*, whilst on the other hand he is certain that an insulated endocarditis can cause it, and that too in a very violent degree.

As to the sounds, normal and abnormal, audible in the arteries, considerable additions to our knowledge have been made since the appearance of Laennec's work. Of the two, it was only those of the abnormal kind which were noticed by this great observer. M. Bouillaud has turned his attention to those existing in the natural state, and has observed that, in all the great arteries of the trunk, neck, and limbs, a kind of low murmur accompanies each wave of the pulse, varying in character according to the size of the artery, the rapidity of the contractions of the heart, the age of the subject, &c. This sound he compares to that produced by the friction of the points of the fingers against each other in the action of giving a fillip. If the stethoscope be made to press somewhat more firmly on the artery, this becomes immediately converted into a bellows sound like that heard in narrowing of the orifices of the heart, or the "*bruit placentaire*," audible in the abdomen in pregnancy. M. Laennec has strongly asserted that such sounds are peculiar to the arteries of hypochondriacal patients. M. Bouillaud fancies that, in proportion as the vessels are soft and relaxed, and contain less than their due quantity of blood, or if the fluid is thin and watery, the rushing sound becomes less obscure, and approximates more to the bellows sound. The arterial sound is single, instead of being double, like that of the heart, and is synchronous near this organ with the first of the two cardiac sounds; whilst, in the more distant arteries, it is, as we have seen with regard to the arterial impulse, slightly posterior to it, as becomes quite evident in cases where the pulse is very slow, below fifty, for example. That the arterial sound depends on the friction of the column of blood is obvious, as it can easily be imitated by injecting a fluid into the arteries of the dead subject, or through any elastic tubes. M. Piorry's experiments on this point in the "Archives Générales" have thrown some light on the subject . The bellows sound, according to him, may exist without any narrowing of the arterial tube, though the existence of this will certainly much increase its intensity. The passage of the blood through an aneurismal tumour presents this phenomenon in a very marked degree. M. Pelletan is in opposition with M. Piorry in relation to the cause of this sound; for he conceives that some degree either of roughness or of narrowing, or some other obstacle in the tube, is essentially necessary to the production of such sounds; but our author is far from coinciding with him in these opinions. Dr. Corrigan, in his paper on what he supposes to be a newly discovered disease of the heart (permanent patency of the aortic valves,) has observed that, if a slight pressure be made on a flexible tube, a "*bruit de soufflet*" and "*frémissement*" exist to a certain distance beyond such pressure, that is, in that portion, which, from the limited quantity of fluid admitted, is no longer tense; and the intensity of these phænomena varies with the rapidity of the current.

According to M. Bouillaud, the abnormal sounds of the arteries are divisible into four species: viz. the ordinary intermittent bellows sound; the continuous bellows sound, (*bruit de soufflet à double courant*); the "*bruit ou ronflement du diable;* and, finally, the "*bruit musical,*" or modulated variety, resembling the simplest form of a musical air. The first, or ordinary kind, is synchronous with the arterial diastole, and occasionally accompanied by *frémissement cataire:* and, as already mentioned, is producible by pressure, from whatever cause. Hence the development of a tumour in the neighbourhood of a large artery is often sufficient to give rise to it. From this source he has met with it in the iliac artery of a female, and to such a degree as accurately to imitate the placentary murmur of pregnancy; and even the latter phenomenon he attributes to a similar cause. Of the continuous bellows sound, including the "*bruit ou ronflement du diable,*" which is the most curious variety of it, he has met within the last three years, or since his attention became directed to the subject, a great number of instances. He has been induced to give this strange name to the variety of it just mentioned, from its close resemblance to the sound produced by the toy called the devil on two sticks, which was in fashion a few years since, both in France and this country, consisting of a couple of small humming tops placed end to end, and which on a rotatory movement being given it, in tossing it up into the air, produces a whizzing or booming sound. This species of soufflet is, in short, the bellows sound in its highest degree of intensity, and is sometimes compared by our author to that produced by the great bellows of a forge. The arterial sound sometimes imitates the cooing of a wood pigeon; at others the whistling of the wind through a keyhole or other small cleft. The arteries in which the "*bruit du diable*" are most frequently met with are the carotid and subclavian. Although continuous and uniform in appearance, yet, with a little attention, we can easily satisfy ourselves that it runs up through a kind of scale, the lowest sounds of which may be compared to the bellows of a smith's forge, and the highest and most acute to the instrument above alluded to. It may be considerably modified by making pretty firm pressure with the stethoscope on the artery, being thus in some cases much weakened, and in others converted into a low grumbling character, or into a murmuring sound like that heard on applying a large shell to the ear, or like the confused hum of a crowd. Even a slight change of position in the patient's head will occasionally greatly alter its intensity. Thus, in general, it increases in strength when the hand is carried to the opposite side from that of the artery under examination, and the chin is a little raised; and, what is very singular, if we push the larynx away from the sonorous artery, the sound immediately diminishes, or even ceases altogether; and also, as M. Donné has observed, if the patient makes any prolonged muscular effort, it is likewise at an end. The musical variety has been already well described by Laennec, and even expressed by him in musical notation. It occasionally resembles the humming of winged insects or the sound of the jews' harp. It occurs most frequently in chlorotic cases; such as, from the existence of palpitations and oppression on the slightest exercise, &c. are often mistaken for organic disease of the heart. In fact, M. Bouillaud looks upon these sounds as an invariable accompaniment of this morbid state of the constitution. The patients who are the subjects of it are remarkably pale and delicate in appearance, and of a very nervous habit; and he seems to think it may here have some connexion with the unnatural predominance of the serum in the blood in such individuals, and the consequently thin and watery condition of this fluid. This, however, appears to us far from certain.

The causes of all these varieties of arterial sounds are reducible to the following heads: 1st, Compression, as by a tumour, the stethoscope, &c. 2d, Contraction of the caliber of the artery, whether by organic lesion or any other cause. 3d, Roughness of the lining membrane, from osseous degeneration, &c. 4th, Violent action of the left ventricle of the heart. 5th, The passage of the blood through an accidental opening in an artery, and its mixture with the blood in the vein, (aneurismal varix.) According to Dr. Corrigan, to these should be added flaccidity of the arterial walls; but this cause is rejected by our author, who at the same time takes occasion to question the novelty of the disease described by the writer just named, under the title of permanent patency of the mouth of the aorta, or the inefficiency of the aortic valves; one of the most conspicuous symptoms of which is stated to be a *bruit de soufflet*, in the ascending aorta, the carotids, and subclavians; a phenomenon which is ascribed to the reflux of the blood into the ventricles after each systole, causing flaccidity of the aorta and great branches arising from it, so that the next wave of blood, by its motion under these circumstances, gives rise to a vibration in the walls of the arteries, and to a consequent bellows sound, synchronous with their diastole. M. Bouillaud attempts to throw discredit, though we think very unreasonably, on the reality of the experiments adduced to prove directly that this sound depends on flaccidity of the arteries; and asserts that it is in contradiction with all others on the same subject, and especially with that where the pressure of the stethoscope gives rise to this phenomenon. We do not, however, we confess, see the apposition between the two facts; for pressure on any point of a flexible tube filled with a fluid tends necessarily, by suffering a smaller quantity of the contained fluid to pass than what is requisite to distend it, to produce a state of relative relaxation in the portions immediately beyond that where such pressure is applied; and that this want of tension in the arteries may be productive of a sound like that in question, appears to receive support from the following passage in the work of M. Bouillaud before us, when speaking of a case in which the "*bruit du diable*" and "*frémissement cataire*" were very evident in the right carotid and subclavian: he says, it seemed as if these arteries were not sufficiently full, and as if the molecules of the blood, in consequence of this, came into collision with each other. M. Guyot, though he considers himself a partisan of Dr. Corrigan's opinion, has rather thrown a difficulty in the way of its adoption, by asserting that the soufflet in question is synchronous, not with the diastole of the arteries, but with that of the ventricles, and consequently with the arterial systole; and he ascribes it to the friction of the blood, in its retrograde course, against the edges of the diseased sigmoid valves, as well as against the sides of the artery. In regard to this, however, M. Bouillaud asserts that it is not merely during the arterial systole that the soufflet is heard in such cases, but also, and principally, during the arterial diastole; that is, in the instant of the ventricular contraction. The possibility of this double soufflet existing in such cases was known also to Dr. Corrigan, as he states expressly that, when the deficiency of the valves is considerable, allowing the blood to rush back in a full stream into the ventricle, two such sounds exist, the first corresponding with the diastole of the artery, and resembling a rushing of blood into the aorta; the second, which immediately succeeds, seeming to the ear like a rushing back of the blood into the ventricle. The primary element in the production of these sounds M. Bouillaud conceives to be friction against the inner surface of the arteries, which are often also remarkably modified as to their vibratory power by variations in their tension and volume, and in the thickness of their walls, and also by changes in the quality of the blood circulating through them. With regard to the infinitely greater frequency of the "*bruit du diable*" in the carotids and subclavians, he discusses the point as to whether it may not depend on their proximity to the heart, and the greater impulse of the blood to which they are consequently exposed; and also suggests the possibility of its greater intensity here being connected with the neighbourhood of the larynx and trachea, which he conceives may, like the sounding board of a musical instrument, tend to multiply the tone. Any sudden effort, accompanied by closure of the larynx, and consequent cessation of the vibration of the enclosed air, certainly interrupts it; but whether this may not have some connexion with the pressure made on the arteries by the action of the adjacent muscles is dubious.

As the nature, causes, and practical value of the sounds produced near the impregnated uterus, and by the motion of the fœtal heart, have been fully discussed in other articles, we shall pass over entirely M. Bouillaud's valuable chapter on this subject; observing merely, that our author is the great advocate of the doctrine which refers the sound termed *placental* to the compression of the hypogastric arteries by the gravid uterus.

The next portion of the work consists of some general considerations of the diseases of the heart, and is followed by a very detailed account of the particular affections to which it is liable, illustrated by numerous cases, many of which are of great interest. Of these it is our intention to present our readers with a pretty full analysis; but the length to which the present article has run obliges us to defer the fulfilment of our design to the next Number.

---

